# Microhardness and characterization of human dental tissue after application of enzymatic chemical agents: *In vitro* study

**DOI:** 10.4317/jced.61811

**Published:** 2024-08-01

**Authors:** Caio-Luiz Lins-Candeiro, Wender Batista-de-Souza, Murilo Navarro-de-Oliveira, Paulo-César-Freitas Santos-Filho, Luiz-Renato Paranhos

**Affiliations:** 1 PhD student, Postgraduate program in dentistry, Faculty of Dentistry, Federal University of Uberlândia; 2 MSc student, Postgraduate program in dentistry, Faculty of Dentistry, Federal University of Uberlândia; 3 Faculty of Dentistry, Centro Universitário das Faculdades Associadas de Ensino; 4 Department of Operative Dentistry and Dental Materials, Faculty of Dentistry, Federal University of Uberlândia; 5 Department of Preventive and Social Dentistry, Faculty of Dentistry, Federal University of Uberlândia

## Abstract

**Background:**

The use of enzymatic chemical agents are papain-based materials used in the selective removal of decayed dentin tissue, assisting in conservative techniques and reducing the chances of accidental pulp exposure.

**Material and Methods:**

A research protocol was subjected to and approved by an Ethics Committee. Using a reporting guide for laboratory studies (CRIS). Healthy human teeth comprised the produced dentin discs subjected to polishing and washing in an ultrasonic bath. Next, the discs received material application according to the experimental groups: water-soluble gel for two minutes, 37% phosphoric acid for 15 seconds in dentin and 30 seconds in enamel, Papacárie Duo (PD) for 30 seconds and two minutes, and Brix 3000 (BX) for two minutes and 30 seconds. The measurement of material pH used solutions at concentrations of 0.1 ml and 2.7 ml prepared for each enzymatic agent. Then, a bench pH meter (n=10) and pH indicator strips determined pH values. The discs underwent the Knoop hardness test (n=10). The sample calculation was performed using the GPower software with α = 0.05, effect 0.63 and power of 95%. Descriptive statistical analysis was carried out for pH, one way ANOVA supplemented by Tukey for knoop hardness and Spearman correlation for pH measurement techniques.

**Results:**

The enamel hardness findings indicated that, after material application, ECAs do not statistically differ from water-soluble gel (*p*<0.05). The dentinal hardness analysis presented a statistical difference in phosphoric acid from the other groups (*p*<0.05). In the pH test, BX values were lower (4.37 ± 0.01) than PD (4.85 ± 0.06). The groups statistically differed (*p*<0.05).

**Conclusions:**

ECAs for removing decayed dentin tissue did not significantly alter the hardness of enamel and dentin, removal of the smear layer is time-dependent and presents acidity.

** Key words:**Dental caries, Hardness, Dental materials, Papain.

## Introduction

Dental caries is a multifactorial and non-communicable disease mediated by biofilm and modulated by diet, causing mineral losses from hard dental tissues (1). It usually originates from the action of microorganisms in oral microbiota and simple carbohydrate fermentation, mainly sucrose (2).

As for the treatment of cavitated carious lesions, the non-selective removal of carious tissues has long been advocated, completely removing the affected and contaminated tissue in surrounding and pulpal walls, and this is the technique most used by dentists (3,4). However, selective caries removal significantly reduced the risk of pulp exposure, symptoms, and postoperative pulpal complications (5). Thus, selectively removing the carious tissues showed a higher cost-effectiveness than non-selective removal, favoring the vitality maintenance of deeply decayed teeth (6,7).

Brix 3000 is a new papain-based material with higher enzyme concentrations that presents favorable characteristics, such as lower cytotoxicity and genotoxicity levels and faster carious tissue removal (8,9). The amount of papain in its formulation (3000 U/mg at 10%) distinguishes this material. It also uses the EBE (Encapsulating Buffer Emulsion) technology with encapsulated papain, increasing enzymatic activity and providing higher stability.

Numerous studies have been developed after introducing enzymatic chemical agents (ECAs) for selective caries removal. The publications include studies assessing the activity of these ECAs on cells (10), especially pulp cells, analyzing their direct (9) and indirect (11) cytotoxicity, genotoxicity (8), bioactivity (12), and comfort in controlling the painful experience without local anesthetics (13). Other studies assessed the effect of dentin deproteinization caused by chemo-mechanical removers on self-etch adhesive sealing (14,15).

Therefore, the topic has been investigated, increasingly promoting discussions in the literature. However, data regarding ECA action in mineralized dental tissues, such as enamel and dentin, are still scarce. Therefore, the present study assessed surface structure, enamel and dentin hardness, and the pH behavior of these materials after applying enzymatic chemical removers.

## Material and Methods

-Protocol and ethical criteria

It is a controlled *in vitro* study. The researchers complied with ethical criteria related to studies in human beings (CAAE 28490619.3.0000.5152) and followed the CRIS (Checklist for Reporting In-vitro Studies) reporting guide.

-Sample calculation

Gpower 3.1.9.2 software assisted sample calculation using the parameters α = 0.05, 0.63 effect, and 95% power. Therefore, the sample size included 60 samples (n=10). Pilot trials determined sample parameters. The sample size calculation was based on the study outcomes, namely the Knoop microhardness of dental tissues.

-Sample preparations

The study used healthy human third molars donated after exodontia with clinical and radiographic indication. Immediately after exodontia, the teeth were cleaned with periodontal curettes and stored in distilled water under refrigeration in a glass recipient hermetically sealed.

The teeth were sectioned in a cutting machine (Isomet1000; Buehler Ltd, Lake Bluff, IL) with a diamond disc (Diamond Wafering Blade. #3041201 - 4” x.012 X 1/2” - 102 mm x 0.3 mm x 127 mm Odeme Dental Research, Miami, FL, USA) in the buccolingual direction, leveling out the occlusal surface. Later, a new section was made, producing dentin and enamel discs with 2 mm of thickness. Seventy-eight discs were obtained (only one per tooth) so that all would maintain the occlusal-cervical thickness dimensions. The study did not use teeth with carious lesions, anomalies, cracks, fractures, restorations, and discs with pulp horn exposure. The discs were stored in microtubes for up to 30 days in distilled water and constant refrigeration.

The discs underwent a surface polishing protocol with 400, 600, 1200, and 1500-grit sandpapers for 10 seconds each, with a metallic device (50 g) in constant water refrigeration. Next, the discs were washed in an ultrasonic bath (Cristófoli Equipamentos de Biossegurança LTDA, Campo Mourão – PR, Brazil) with distilled water for two five-minute cycles, leaving the polished surface of the disc upward (11).

-Material application protocol

Regarding material application, the samples were randomly distributed into six groups according to the materials: water-soluble gel for two minutes, 37% phosphoric acid for 15 seconds in dentin and 30 seconds in enamel, Papacárie Duo (PD) for 30 seconds and two minutes, and Brix 3000 (BX) for two minutes and 30 seconds. Insulin syringes applied the materials to standardize their quantity. After material application according to the protocol, the discs were washed in distilled water aided by a triple syringe and dried in sterile cotton.

-Knoop hardness test

The discs were included with crystal resin, and the matrix comprised a polyvinyl chloride pipe with 15 mm of height and 21 mm with utility wax at the base. Tilt during indentations was prevented by leveling out the base with 400-grit sandpapers, and the samples underwent the microhardness test (n=10) with a microhardness tester (FM – 700, Future-Tech Corp., Tokyo, Japan). Three indentations were made per sample with a load of 50 g for 15 seconds, respecting a minimum distance of 100 µm between each indentation for dentin and enamel. The mean length of the two diagonals produced by the indenter calculated hardness values. The results correspond to the means of the three indentations for each sample.

-pH test

Two solutions helped measure hydrogen potential by dissolving 0.1 ml and 0.4 ml of each enzymatic remover into 14 ml of distilled water, obtaining two final solutions with 0.7% and 2.7% concentrations, respectively. Solution homogenization used a sterile Falcon tube where the material was poured with distilled water in a multi-platform agitator (Kasvi® K40-10208) at 1900 rpm. Next, a 24-well plate received the solution, using six wells for each material, with 2 ml of solution per well. Control solutions comprised the Milton solution, distilled water, pure water-soluble gel, and 0.7% phosphoric acid (n=10). A bench pH meter (MS Tecnopon Equip. Especiais LTDA) assessed the pH individually for each well in triplicate. Then, a new assessment was performed using pH indicator strips (Merck KGaA, Darmstadt, Germany). Three operators performed all analyses, tabulating the mean values of each evaluation.

-Scanning electron microscopy

The samples received the material application protocol (n=3) and were dehydrated in alcoholic solutions in an increasing sequence of 50%, 70%, 80%, and 90% for ten minutes in each concentration and absolute alcohol three times for ten minutes each. Then, the discs were fixed in stubs, dried at room temperature in a chapel for 24 hours, maintained for 15 days in a desiccator, and metalized before scanning electron microscopy.

-Statistical analysis

The findings were described and tabulated in Excel software, version 2016 Microsoft Office®. Next, the values underwent descriptive analysis, the Shapiro-Wilk normality test, one-way ANOVA supplemented by Tukey’s test at 5% significance, and the Spearman correlation test in JAMOVI statistical software, version 2.3.21. Qualitative results were described and inserted in the study, complementing the quantitative data.

## Results

-Knoop hardness test

**Figure 1:**
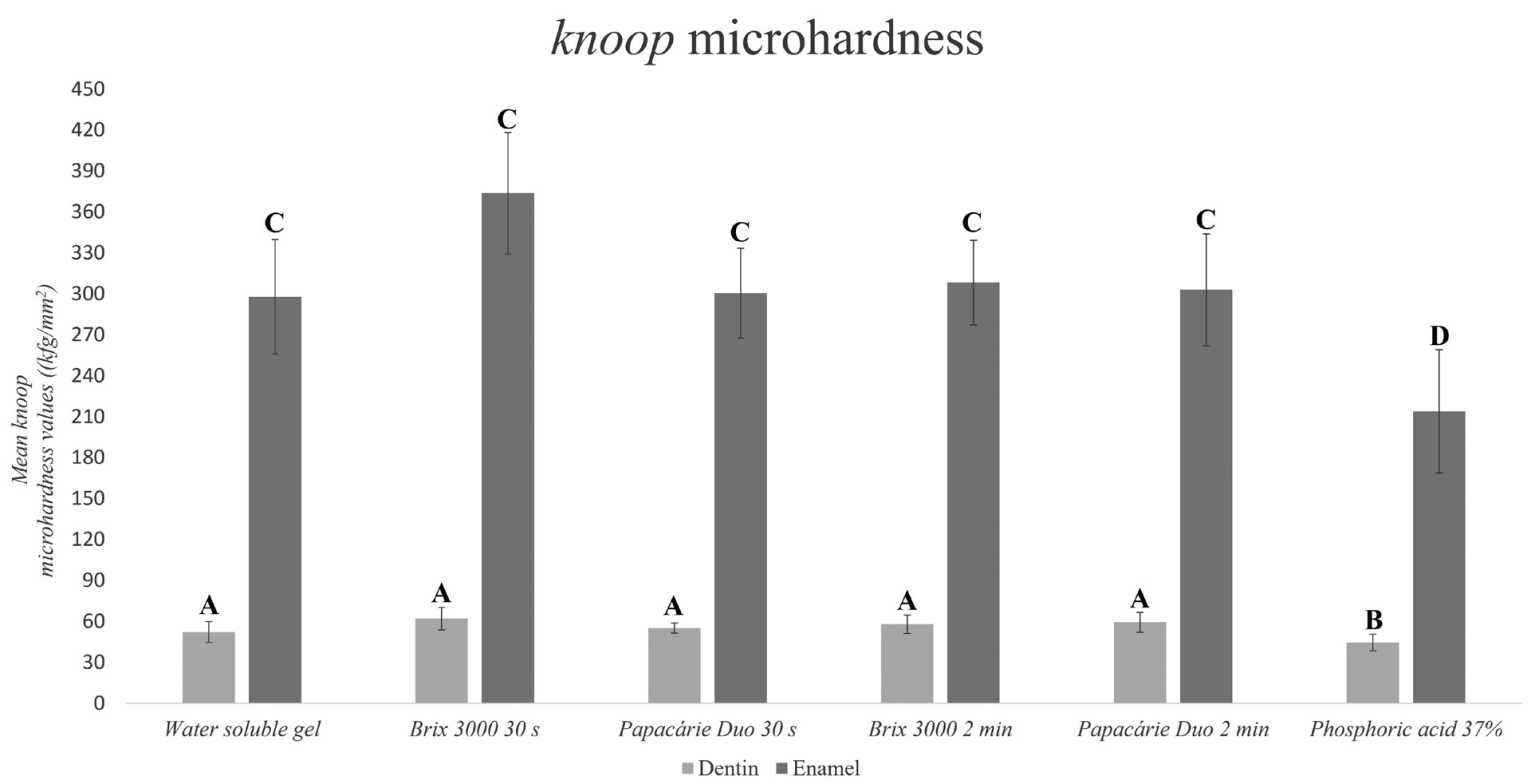
Mean knoop hardness values of human dentin and enamel after applying the experimental materials (n=10). One-way ANOVA supplemented by Tukey’s test at 5% significance, Shapiro-Wilk p=0.513. Different letters indicate statistical differences.

The dentinal hardness analysis (Graph 1) showed that samples receiving phosphoric acid differed statistically from the other groups (*p*<0.05). The knoop microhardness values of tooth enamel (Fig. 1) indicates that, after material application, the samples receiving enzymatic chemical agents (ECAs) did not statistically differ from those receiving the water-soluble gel (*p*<0.05).

-pH test

**Figure 2:**
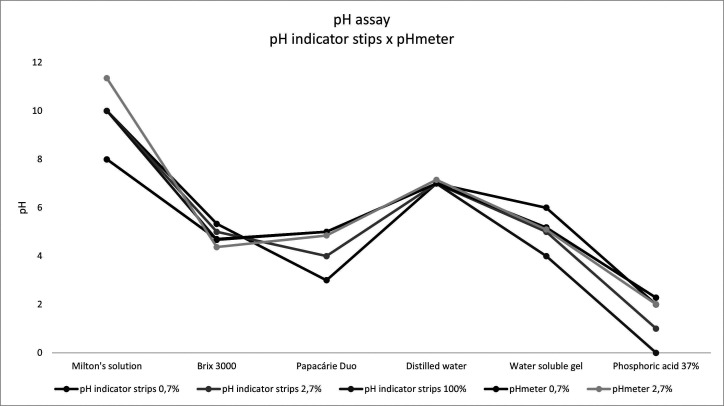
Mean pH values obtained through different techniques: pH indicator strips and bench pH meter.

In the pH test, BX values were lower (4.37 ± 0.01) than PD (4.85 ± 0.06) (Fig. 2). The pH values did not present normal data distribution *p*<0.001) ( Table 1).

**Figure 3:**
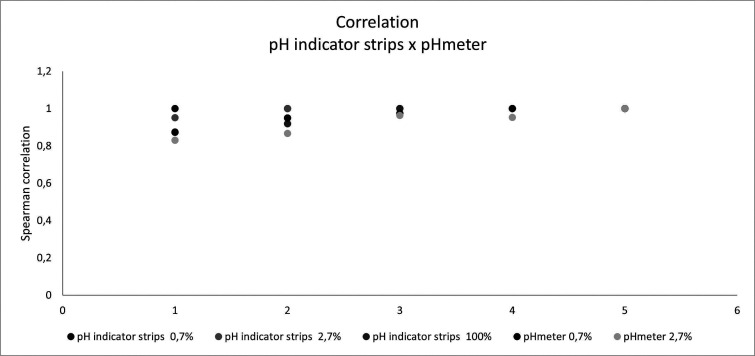
Spearman correlation between different techniques and concentrations (*p*=0.001).

The Shapiro-Wilk normality analysis showed that groups did not present a normal distribution (*p*<0.001); hence, the Spearman test was used. Spearman correlation coefficient demonstrated significance between all groups (*p*=0.001), with positive correlations with a minimum of 0.831 (Fig. 3).

-Scanning electron microscopy

**Figure 4:**
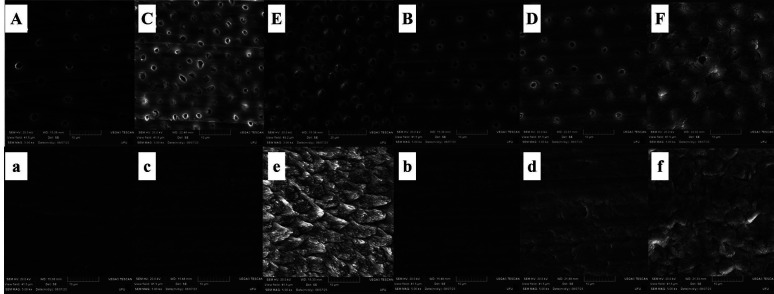
SEM of dentin after the material application protocol. (A) Papacárie Duo for 30 seconds; (B) Papacárie Duo for two minutes; (C) Brix 3000 for 30 seconds; (D) Brix 3000 for two minutes; (E) Phosphoric acid for 15 seconds; (F) Water-soluble gel for two minutes. The SEM of enamel (a) Papacárie Duo for 30 seconds; (b) Papacárie Duo for two minutes; (c) Brix 3000 for 30 seconds; (d) Brix 3000 for two minutes; (e) Phosphoric acid for 15 seconds; (f) Water-soluble gel for two minutes. Magnification of 5000x.

Scanning electron microscopy showed that the longer the contact time of ECAs with dentin (Fig. 4), the higher the exposure of dentinal tubules, although changes in inter- and peritubular dentin are not visible. Enamel images (Fig. 4) demonstrate that Papacárie Duo and Brix 3000 did not significantly change enamel prisms as phosphoric acid. Water-soluble gel images present an irregular surface, possibly due to oily compounds.

## Discussion

Understanding material behavior in dental tissues is essential for clinical practice. This knowledge aids dentists and researchers in direct or indirect operative and rehabilitating decision-making, preserving the dental structure to improve adhesion for the success of the proposed treatment and promoting comfort to patients. The literature has been discussing the application of these materials to people with phobias, challenging behavioral management, and young permanent teeth, showing higher patient acceptance (16).

Chemo-mechanical removal methods have promoted less stressful and painful treatments (17). Methods using enzymatic chemical agents (ECAs) for selectively removing dentinal carious tissue promote lower anxiety than treatments with rotary instruments, as therapies with ECAs were less painful when assessing pain experience (18). Thus, treatments with ECAs are a viable therapeutic proposal, considering patient benefits during treatment associated with the positive effect on healthy dental tissues and the maintenance of viable dental tissues (6).

ECAs did not show significant differences in hardness values compared to the control group, indicating that healthy dentin and enamel presented good quality after applying the materials. The quality of tooth remnants is directly associated with adhesion (19,20). Thus, new studies must be performed to assess more parameters affecting the quantity of tooth remnants after applying these materials.

The microscopy images showed differences in enamel surface between the groups using ECAs and the control, and the former presented an erosion pattern. Systematic reviews with meta-analyses found that erosion did not significantly affect enamel bond strength (21,22). However, erosion causes a rough surface similar to conditions after phosphoric acid etching, potentially benefiting adhesive systems that favor surface erosion (23). Studies assessing dental substrate adhesion after using ECAs are relevant and necessary to substantiate clinical decision-making and restorative surgical protocol.

After ECA applications at different times, an intratubular smear plug appeared with intertubular dentin exposure, while the group that received phosphoric acid showed a complete smear layer removal. Smear layer removal may increase fluid flow on the exposed dentin surface, potentially interfering with postoperative sensitivity and the adhesion process, and moisture control and adhesive system selection will be essential for clinical success (24).

This study is not free of limitations. Considering it is an *in vitro* study, the findings may not be considered for clinical practice because the tests occurred in a controlled environment. Thus, studies assessing the quality of tissues with different adhesive systems and clinical studies evaluating postoperative sensitivity, restoration survival, and restorative materials are relevant to explain ECA use and expand the discussions about applying these protocols to the clinical routine.

## Conclusions

ECAs for carious dentinal tissue removal did not significantly change the hardness of healthy human enamel and dentin despite being acidic. The pH measurement technique was a viable alternative to materials commercially presented in gel, especially when the composition of these materials has pigments.

## Figures and Tables

**Table 1:** Descriptive analysis of pH of different analysis techniques and material concentrations.

Concentrations	Mean (SD)	Variance	Shapiro-Wilk (p)
Technique	Strips 0.7%	5.36 ± 1.94	3.78	<0.001
	Strips 2.7%	5.69 ± 2.87	8.22	<0.001
	Strips 100%	5.11 ± 2.96	8.79	<0.001
	pHmeter 0.7%	5.36 ± 1.94	3.78	<0.001
	pHmeter 2.7%	5.69 ± 2.87	8.22	<0.001

## Data Availability

The datasets used and/or analyzed during the current study are available from the corresponding author.

## References

[1] Pitts NB, Zero DT, Marsh PD, Ekstrand K, Weintraub JA, Ramos-Gomez F (2017). Dental caries. Nat Rev Dis Primers.

[2] Mathur VP, Dhillon JK (2018). Dental Caries: A Disease Which Needs Attention. Indian J Pediatr.

[3] Bjørndal L, Simon S, Tomson PL, Duncan HF (2019). Management of deep caries and the exposed pulp. Int Endod J.

[4] Casagrande L, Seminario AT, Correa MB, Werle SB, Maltz M, Demarco FF (2017). Longevity and associated risk factors in adhesive restorations of young permanent teeth after complete and selective caries removal: a retrospective study. Clin Oral Investig.

[5] Li T, Zhai X, Song F, Zhu H (2018). Selective versus non-selective removal for dental caries: a systematic review and meta-analysis. Acta Odontol Scand.

[6] Barros MMAF, De Queiroz Rodrigues MI, Muniz FWMG, Rodrigues LKA (2020). Selective, stepwise, or nonselective removal of carious tissue: which technique offers lower risk for the treatment of dental caries in permanent teeth? A systematic review and meta-analysis. Clin Oral Investig.

[7] Schwendicke F, Stolpe M, Meyer-Lueckel H, Paris S, Dörfer CE (2013). Cost-effectiveness of one- and two-step incomplete and complete excavations. J Dent Res.

[8] Santos TML, Bresciani E, Matos FS, Camargo SEA, Hidalgo APT, Rivera LML (2020). Comparison between conventional and chemomechanical approaches for the removal of carious dentin: an in vitro study. Sci Rep.

[9] Guedes FR, Bonvicini JF, de Souza GL, da Silva WH, Moura CC, Paranhos LR (2021). Cytotoxicity and dentin composition alterations promoted by different chemomechanical caries removal agents: A preliminary in vitro study. J Clin Exp Dent.

[10] AlHumaid J (2020). Efficacy and Efficiency of Papacarie versus Conventional Method in Caries Removal in Primary Teeth: An SEM Study. Saudi J Med Med Sci.

[11] Lins-Candeiro CL, Paranhos LR, de Oliveira Neto NF, Ribeiro RAO, de-Souza-Costa CA, Guedes FR (2024). Viability and oxidative stress of dental pulp cells after indirect application of chemomechanical agents: An in vitro study. Int Endod J.

[12] Maru V, Madkaikar M, Shabrish S, Kambli P, Dalvi A, Setia P (2022). Evaluation and comparison of cytotoxicity and bioactivity of chemomechanical caries removal agents on stem cells from human exfoliated deciduous teeth. Eur Arch Paediatr Dent.

[13] Yun J, Shim YS, Park SY, An SY (2018). New treatment method for pain and reduction of local anesthesia use in deep caries. J Dent Anesth Pain Med.

[14] Kusumasari C, Abdou A, Tichy A, Hatayama T, Hosaka K, Foxton RM (2020). Effect of smear layer deproteinization with chemo-mechanical caries removal agents on sealing performances of self-etch adhesives. J Dent.

[15] Kusumasari C, Abdou A, Nakajima M, Tagami J (2021). Deproteinization of caries-affected dentin with chemo-mechanical caries removal agents and its effect on dentin bonding with self-etch adhesives. J Dent.

[16] Cardoso M, Coelho A, Lima R, Amaro I, Paula A, Marto CM (2020). Efficacy and Patient's Acceptance of Alternative Methods for Caries Removal-a Systematic Review. J Clin Med.

[17] Pascareli-Carlos AM, Martins LF, Silva Gonçalves MD, Pettorossi Imparato JC, Tedesco TK (2021). Pain perception of children after restorative treatments: Atraumatic restorative treatment versus chemomechanical removal - A noninferiority randomized clinical trial. J Indian Soc Pedod Prev Dent.

[18] Ladewig NM, Tedesco TK, Gimenez T, Braga MM, Raggio DP (2018). Patient-reported outcomes associated with different restorative techniques in pediatric dentistry: A systematic review and MTC meta-analysis. PLoS One.

[19] Sauro S, Faus-Matoses V, Makeeva I, Nuñez Martí JM, Gonzalez Martínez R, García Bautista JA (2018). Effects of Polyacrylic Acid Pre-Treatment on Bonded-Dentine Interfaces Created with a Modern Bioactive Resin-Modified Glass Ionomer Cement and Subjected to Cycling Mechanical Stress. Materials (Basel).

[20] Rossoni NB, Cavalheiro CP, Casagrande L, Lenzi TL (2022). Influence of the chemomechanical and mechanical carious tissue removal on the risk of restorative failure: a systematic review and meta-analysis. Clin Oral Investig.

[21] Solon-de-Mello M, da Silva Fidalgo TK, Dos Santos Letieri A, Masterson D, Granjeiro JM, Monte Alto RV (2019). Longevity of indirect restorations cemented with self-adhesive resin luting with and without selective enamel etching. A Systematic review and meta-analysis. J Esthet Restor Dent.

[22] Wiegand A, Lechte C, Kanzow P (2021 Dec). Adhesion to eroded enamel and dentin: systematic review and meta-analysis. Dent Mater.

[23] Erickson RL, Barkmeier WW, Kimmes NS (2009 Oct). Bond strength of self-etch adhesives to pre-etched enamel. Dent Mater.

[24] Firouzmandi M, Khashaei S (2020). Knoop Hardness of Self-Etch Adhesives Applied on Superficial and Deep Dentin. J Dent (Shiraz).

